# Psychotic episode with onset in childhood and adolescence – Factors which may influence the outcome

**DOI:** 10.1192/j.eurpsy.2022.1113

**Published:** 2022-09-01

**Authors:** C.-E. Stoica, A. Iotu, I. Nicolau, C. Nistor, I. Grigore, F. Rad

**Affiliations:** “Prof. Dr. Al. Obregia” Psychiatry Hospital, Child And Adolescent Psychiatry, Bucharest, Romania

**Keywords:** psychotic episode, comorbidities, outcome

## Abstract

**Introduction:**

A psychotic episode might stem from various psychiatric disorders, such as Major Depressive Disorder, Mania, Autism Spectrum Disorder, it might lead to Schizophrenia, or it might be a single event.

**Objectives:**

The study aimed to assess the main comorbidities encountered in the onset of psychotic episodes in children and adolescents, who were hospitalized in a pediatric psychiatry department for at least one night. Furthermore, another objective was to establish whether a family history of mental illness or a poor socio-economic status have bigger impact on the evolution of these patients.

**Methods:**

To analyze the objectives, it was used an observational study, based on patients with the onset of a psychotic episode and associated diagnosis according to ICD-10, evaluated in Child and Adolescent Psychiatric Department of “Prof. Dr. Al. Obregia” Hospital, between 2015-2019. Patients with psychotic episodes with onset related to Major Depressive Disorder and Mania or a personal history of Schizophrenia were excluded.

**Results:**

The most frequent associated comorbidity was Autism Spectrum Disorder. In terms of long-term evolution, patients with comorbidities have poorer outcomes, more relapses and hospitalizations. Family history of mental illness, low socio-economic status, the age of onset were found to be prognostic factors and have an important impact on the outcome.

**Conclusions:**

This study compared patients with psychotic episodes with and without comorbid conditions associated, considering the length of hospitalization period, the evolution and the number of relapses. The presence and the type of comorbidities are important factors of evolution and prognostic for these patients.

**Disclosure:**

No significant relationships.

